# Gene profiling, biomarkers and pathways characterizing HCV-related hepatocellular carcinoma

**DOI:** 10.1186/1479-5876-7-85

**Published:** 2009-10-12

**Authors:** Valeria De Giorgi, Alessandro Monaco, Andrea Worchech, MariaLina Tornesello, Francesco Izzo, Luigi Buonaguro, Francesco M Marincola, Ena Wang, Franco M Buonaguro

**Affiliations:** 1 Molecular Biology and Viral Oncogenesis & AIDS Refer. Center, Ist. Naz. Tumori "Fond. G. Pascale", Naples - Italy; 2 Department of Chemistry, University of Naples "Federico II", Naples, Italy; 3 Infectious Disease and Immunogenetics Section (IDIS), Department of Transfusion Medicine, Clinical Center and Trans-NIH Center for Human Immunology (CHI), National Institutes of Health, Bethesda, MD -USA; 4 Genelux Corporation, Research and Development, San Diego Science Center, San Diego, CA, USA; 5 Department of Biochemistry, Biocenter, University of Wuerzburg, Am Hubland, Wuerzburg, Germany; 6 Div. of Surgery "D", Ist. Naz. Tumori "Fond. G. Pascale", Naples - Italy

## Abstract

**Background:**

Hepatitis C virus (HCV) infection is a major cause of hepatocellular carcinoma (HCC) worldwide. The molecular mechanisms of HCV-induced hepatocarcinogenesis are not yet fully elucidated. Besides indirect effects as tissue inflammation and regeneration, a more direct oncogenic activity of HCV can be postulated leading to an altered expression of cellular genes by early HCV viral proteins. In the present study, a comparison of gene expression patterns has been performed by microarray analysis on liver biopsies from HCV-positive HCC patients and HCV-negative controls.

**Methods:**

Gene expression profiling of liver tissues has been performed using a high-density microarray containing 36'000 oligos, representing 90% of the human genes. Samples were obtained from 14 patients affected by HCV-related HCC and 7 HCV-negative non-liver-cancer patients, enrolled at INT in Naples. Transcriptional profiles identified in liver biopsies from HCC nodules and paired non-adjacent non-HCC liver tissue of the same HCV-positive patients were compared to those from HCV-negative controls by the Cluster program. The pathway analysis was performed using the BRB-Array- Tools based on the "Ingenuity System Database". Significance threshold of *t*-test was set at 0.001.

**Results:**

Significant differences were found between the expression patterns of several genes falling into different metabolic and inflammation/immunity pathways in HCV-related HCC tissues as well as the non-HCC counterpart compared to normal liver tissues. Only few genes were found differentially expressed between HCV-related HCC tissues and paired non-HCC counterpart.

**Conclusion:**

In this study, informative data on the global gene expression pattern of HCV-related HCC and non-HCC counterpart, as well as on their difference with the one observed in normal liver tissues have been obtained. These results may lead to the identification of specific biomarkers relevant to develop tools for detection, diagnosis, and classification of HCV-related HCC.

## Introduction

Hepatocellular carcinoma (HCC) is the most common liver malignancy as well as the third and the fifth cause of cancer death in the world in men and women, respectively [1-3]. As for other types of cancer, the etiology and pathogenesis of HCC is multifactorial and multistep [4]. The main risk factor for development of HCC are the hepatitis B and C virus (HBV and HCV) infection [5-8]. Non viral causes, such as toxins and drugs (i.e., alcohol, aflatoxins, microcystin, anabolic steroids), metabolic liver diseases (i.e., hereditary haemochromatosis, α1-antitrypsin deficiency), steatosis and non-alcoholic fatty liver diseases as well as diabetes, play a role in a minor number of cases [9-11]. The prevalence of HCC in Italy, and in Southern Italy in particular, is significantly higher compared to other Western countries. Hepatitis virus infection, long-term alcohol and tobacco consumption account for 87% of HCC cases in Italian population and, among these, 61% of HCC are attributable to HCV. In particular, a recent seroprevalence surveillance study conducted in the general population of Southern Italy Campania Region reported a 7.5% positivity for HCV infection which peaked at 23.2% positivity in the 65 years or older age group [12]. The multistep progression to HCC, in particular the one associated to hepatitis virus, is characterized by a process including chronic liver injury, tissue inflammation, cell death, cirrhosis, regeneration, DNA damage, dysplasia and finally, HCC. In this multistep process, the cirrhosis represents the preneoplastic stage showing regenerative, dysplastic as well as HCC nodules [13].

The precise molecular mechanism underlying the progression of chronic hepatitis viral infections to HCC is currently unknown. Activation of cellular oncogenes, inactivation of tumor suppressor genes, overexpression of growth factors, telomerase activation and defects in DNA mismatch repair may contribute to the development of HCC [14-16]. In this framework, differential gene expression patterns accompanying different stages of growth, disease initiation, cell cycle progression, and responses to environmental stimuli provide important clues to this complex process.

DNA microarray enables investigators to study expression profile and activation of thousands of genes simultaneously. In particular, the identification of cancer-related stereotyped expression patterns might allow the elucidation of molecular mechanisms underlying cancer progression and provides important molecular markers for diagnostic purposes. This strategy has been recently used to profile global changes in gene expression in liver samples obtained from patients with HCV-related HCC [17-19]. Several of these studies identified gene sets that may be useful as potential microarray-based diagnostic tools. However, the direct or indirect HCV role in HCC pathogenesis is still a controversial issue and additional efforts need to be made aimed to specifically dissect the relationship between stages of HCV chronic infection and progression to HCC.

The present study has been focused on investigating genes and pathways involved in viral carcinogenesis and progression to HCC in HCV-chronically infected patients.

## Materials and methods

### Patient and Tissue Samples

Liver biopsies from fourteen HCV-positive HCC patients and seven HCV-negative non-liver cancer control patients (during laparoscopic cholecystectomy) were obtained with informed consent at the liver unit of the INT "Pascale" in Naples. In particular, from each of the HCV-positive HCC patients, a pair of liver biopsies from HCC nodule and non-adjacent non-HCC counterpart were surgically excised. All liver biopsies were stored in RNA Later at -80°C (Ambion, Austin, TX). Confirmation of the histopathological nature of the biopsies was performed by the Pathology lab at INT before the processing for RNA extraction. The non-HCC tissue from HCV-positive patient were an heterogeneous sample representing the prevalent liver condition of each subject (ranging from persistent HCV-infection to cirrhotic lesions). Furthermore, laboratory analysis confirmed that the 7 controls were seronegative for hepatitis C virus antibodies (HCV Ab).

### Preparation of RNA, probe preparation, and microarray hybridization

Samples were homogenized in disposable tissue grinders (Kendall, Precision). Total RNA was extracted by TRIzol solution (Life Technologies, Rockville, MD), and purity of the RNA preparation was verified by the 260:280 nm ratio (range, 1.8-2.0) at spectrophotometric reading with NanoDrop (Thermo Fisher Scientific, Waltham, MA). Integrity of extracted RNA was evaluated by Agilent 2100 Bioanalyzer (Agilent Technologies, Palo Alto, CA), analyzing the presence of 28S and 18S ribosomal RNA bands as well as the 28S/18S rRNA intensity ratio equal or close to 1.5. In addition, phenol contamination was checked and a 260:230 nm ratio (range, 2.0-2.2) was considered acceptable.

Double-stranded cDNA was prepared from 3 μg of total RNA (T-RNA) in 9 μl DEPC -treated H_2_O using the Super script II Kit (Invitrogen) with a T7-(dT15) oligonucleotide primer. cDNA synthesis was completed at 42°C for 1 h. Full-length dsDNA was synthesized incubating the produced cDNA with 2 U of RNase-H (Promega) and 3 μl of Advantage cDNA Polymerase Mix (Clontech), in Advantage PCR buffer (Clontech), in presence of 10 mM dNTP and DNase-free water. dsDNA was extracted with phenol-chloroform-isoamyl, precipitated with ethanol in the presence of 1 μl linear acrylamide (0.1 μg/μl, Ambion, Austin, TX) and aRNA (amplified-RNA) was synthesized using Ambion's T7 MegaScript in Vitro Transcription Kit (Ambion, Austin, TX). aRNA recovery and removal of template dsDNA was achieved by TRIzol purification. For the second round of amplification, aliquots of 1 μg of the aRNA were reverse transcribed into cDNA using 1 μl of random hexamer under the conditions used in the first round. Second-strand cDNA synthesis was initiated by 1 μg oligo-dT-T7 primer and the resulting dsDNA was used as template for in vitro transcription of aRNA in the same experimental conditions as for the first round [20]. 6 μg of this aRNA was used for probe preparation, in particular test samples were labeled with USL-Cy5 (Kreatech) and pooled with the same amount of reference sample (control donor peripheral blood mononuclear cells, PBMC, seronegative for hepatitis C virus antibodies (HCV Ab)) labeled with USL-Cy3 (Kreatech). The two labeled aRNA probes were separated from unincorporated nucleotides by filtration, fragmented, mixed and co-hybridized to a custom-made 36 K oligoarrays at 42°C for 24 h. The oligo-chips were printed at the Immunogenetics Section Department of Transfusion Medicine, Clinical Center, National Institutes of Health (Bethesda, MD). After hybridization the slides were washed with 2 × SSC/0.1%SDS for 1 min, 1 × SSC for 1 min, 0.2 × SSC for 1 min, 0.05 × SSC for 10 sec., and dried by centrifugation at 800 g for 3 minutes at RT.

### Data Analysis

Hybridized arrays were scanned at 10-μm resolution with a GenePix 4000 scanner (Axon Instruments) at variable photomultiplier tube (PMT) voltage to obtain maximal signal intensities with less than 1% probe saturation. Image and data files were deposited at microarray data base (mAdb) at  and retrieved after median centered, filtering of intensity (>200) and spot elimination (bad and no signal). Data were further analyzed using Cluster and TreeView software (Stanford University, Stanford, CA).

### Statistical Analysis

#### Unsupervised Analysis

For this analysis, a low-stringency filtering was applied, selecting the genes differentially expressed in 80% of all experiments with a >3 fold change ratio in at least one experiment. 7'760 genes were selected for the analysis including the three groups of analyzed samples (the HCV-related HCC, their non-HCC counterpart, as well as samples from the controls); 5'473 genes were selected for the analysis including the HCV-related HCC and normal control samples; 6'069 genes were selected for the analysis including the HCV-related non-HCC paired tissue and normal control samples. Hierarchical cluster analysis was conducted on these genes according to Eisen et al. [21]; differential expressed genes were visualized by Treeview and displayed according to the central method [22].

#### Supervised Analysis

Supervised class comparison was performed using the BRB ArrayTool developed at NCI, Biometric Research Branch, Division of Cancer Treatment and Diagnosis. Three subsets of genes were explored. The first subset included genes upregulated in HCV-related HCC compared to normal control samples, the second subset included genes upregulated in the HCV-related non-HCC counterpart compared with normal control samples, the third subset included genes upregulated in HCV-related HCC compared to the non-HCC paired liver tissue samples. Paired samples were analyzed using a two-tailed paired Student's *t*-test. Unpaired samples were tested with a two-tailed unpaired Student's *t*-test assuming unequal variance or with an *F*-test as appropriate. All analyses were tested for an univariate significance threshold set at a *p*-value < 0.01 for the first subset of genes and at a *p*-value < 0.001 for the second subset. Gene clusters identified by the univariate *t*-test were challenged with two alternative additional tests, an univariate permutation test (PT) and a global multivariate PT. The multivariate PT was calibrated to restrict the false discovery rate to 10%. Genes identified by univariate *t*-test as differentially expressed (*p*-value < 0.001 and *p*-value < 0.01) and a PT significance <0.05 were considered truly differentially expressed. Gene function was assigned based on Database for Annotation, Visualization and Integrated Discovery (DAVID) and Gene Ontology .

#### Ingenuity pathway analysis

The pathway analysis was performed using the gene set expression comparison kit implemented in BRB-Array- Tools. The human pathway lists determined by "Ingenuity System Database" was selected. Significance threshold of *t*-test was set at 0.001. The Ingenuity Pathways Analysis (IPA) is a system that transforms large data sets into a group of relevant networks containing direct and indirect relationships between genes based on known interactions in the literature.

## Results

### Quality Control

**Figure 1:**
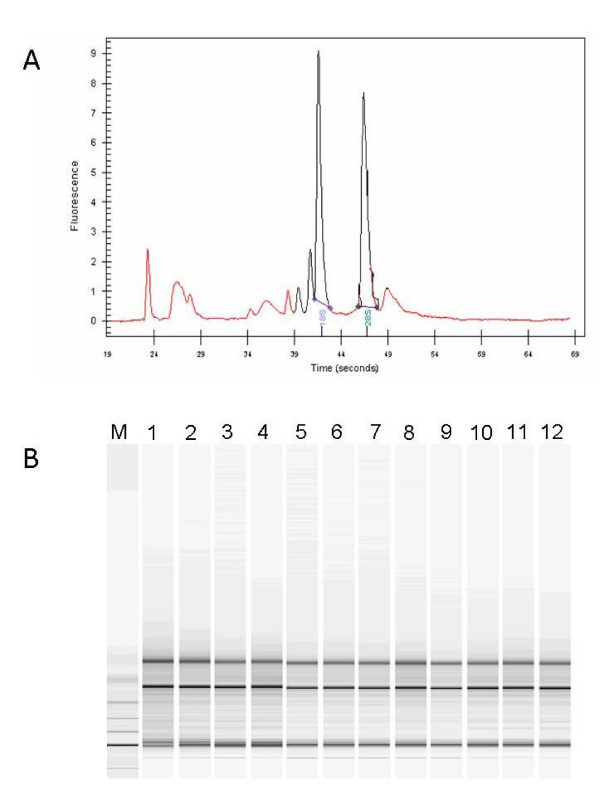
**Purity and integrity quality control of total extracted RNA**. (A) Representative Electropherogram of total RNA extracted from samples included in the analysis. (B) Representative Gel image evaluation of RNA integrity and 28S/18S rRNA ratio.

The quality of extracted total RNA was verified by Agilent 2100 Bioanalyzer (Agilent Technologies, Palo Alto, CA), showing discrete 28S and 18S rRNA bands (Figure 1A) as well as a 28S/18S rRNA intensity ratio equal or close to 1.5 which is considered appropriate for total RNA extracted from liver tissue biopsies ("Assessing RNA Quality", ). Based on this parameter, all extracted total RNA samples met the quality control criteria (Figure 1B).

### Unsupervised analysis is concordant with Pathological Classification

**Figure 2:**
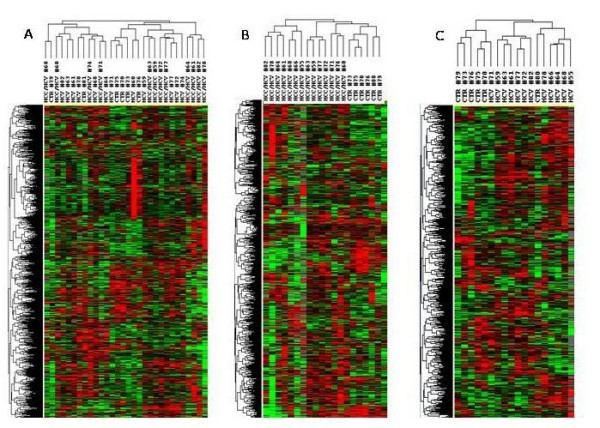
**Unsupervised hierarchical clustering**. Overall patterns of expression of genes across the 14 HCV-related HCC and non-HCC counterpart, as well as 7 HCV-negative control patients. Red indicates over-expression; green indicates under-expression; black indicates unchanged expression; gray indicates no detection of expression (intensity of both Cy3 and Cy5 below the cutoff value). Each row represents a single gene; each column represents a single sample. The dendrogram at the left of matrix indicates the degree of similarity among the genes examined by expression patterns. The dendrogram at the top of the matrix indicates the degree of similarity between samples. Panel A, unsupervised analysis including all three set of samples; Panel B, unsupervised analysis including HCV-related HCC and normal control liver samples; Panel C, unsupervised analysis including HCV-related non-HCC counterpart and normal control liver samples.

The gene expression profiles of tissue samples from the three groups of analyzed samples (the HCV-related HCC, their non-HCC counterpart, as well as samples from control patients) were compared by an unsupervised analysis. No clear separation of the 3 different groups was observed, although control samples clustered mainly with samples from HCV-related non-HCC paired tissue, which includes dysplastic lesion in cirrhotic liver, representing a pre-neoplastic step (Figure 2A).

In order to identify genes differentially modulated in HCV-related lesions compared to normal liver tissue samples, an unsupervised analysis was then performed including only paired samples from HCV-related HCC and normal control samples or from the HCV-related non-HCC counterpart and control samples (Figures 2B and 2C). According to filtering described in Material and Methods, HCV-related HCC and normal control samples showed 5'473 genes differentially expressed, with a perfect clustering according to histological characteristics (Figure 2B). Similarly, HCV-related non-HCC tissue and normal control samples showed 6'069 genes differentially expressed with a perfect clustering according to histological characteristics also in this case (Figure 2C). The only exception to this pattern is represented by the normal control sample (CTR#80) which did not fall in the control cluster (CTR).

### Supervised analysis

The supervised analysis was performed comparing pairs of gene sets using an unpaired Student's *t*-test with a cut-off set at *p *< 0.01.

**Figure 3:**
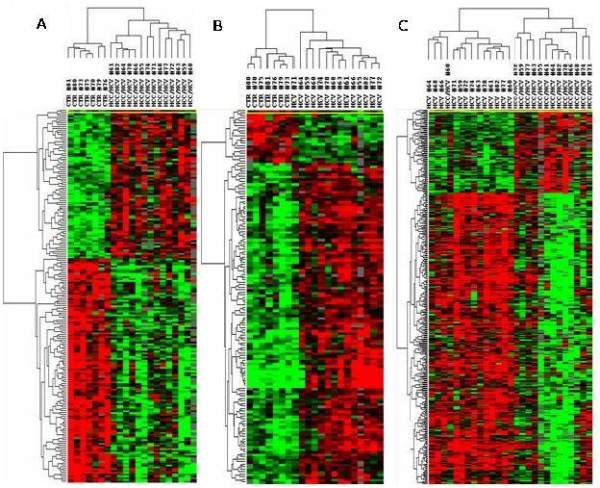
**Heat map of the gene signature, identified by Class Comparison Analysis**. Panel A, analysis including HCV-related HCC and normal control liver samples; Panel B, analysis including HCV-related non-HCC liver tissues and control liver samples; Panel C, analysis including HCV-related HCC and HCV-related non-HCC counterpart liver samples. The expression pattern of the genes is shown each row represents a single gene.

The analysis comparing gene sets in liver tissues from HCV-related HCC and normal controls identified 825 genes differentially expressed. Among them, 465 were shown to be up-regulated and 360 down-regulated in HCV-related HCC liver tissues (Figure 3A). The first 40 genes showing the highest fold of up-regulation are listed in Table 1.

**Table 1:** The first 40 up-regulated genes in HCV-related HCC

**N°**	**Gene Name**	**Description**
1	RYBP	RING1 and YY1 binding protein (RYBP)
2	ATP1B3	ATPase, Na+/K+ transporting, beta 3 polypeptide
3	TMC	transmembrane channel-like 7 (TMC7)
4	ZNF567	zinc finger protein 567 (ZNF567
5	GPR108	G protein-coupled receptor 108 (GPR108), transcript variant 1
6	CD19	CD19 molecule
7	SPINK1	**serine peptidase inhibitor, Kazal type 1**
8	CDC2L6	cell division cycle 2-like 6 (CDK8-like)
9	RSRC1	arginine/serine-rich coiled-coil 1 (RSRC1)
10	METAP	methionyl aminopeptidase 1
11	GPC3	glypican 3
12	SNHG11	Small nucleolar RNA host gene (non-protein coding) 11
13	RY1	putative nucleic acid binding protein RY-1 (RY1)
14	CRELD2	cysteine-rich with EGF-like domains 2 (CRELD2)
15	GLUL	glutamate-ammonia ligase (glutamine synthetase)
16	SERPINB1	serpin peptidase inhibitor, clade B (ovalbumin), member 1 (SERPINB1)
17	TRMT6	tRNA methyltransferase 6 homolog (S. cerevisiae)
18	UNC13D	unc-13 homolog D (C. elegans) (UNC13D)
19	E4F1--E4F	E4F transcription factor 1 (E4F1)
20	SLC22A2	solute carrier family 22 (organic cation transporter), member 2 (SLC22A2)
21	CNIH4	cornichon homolog 4 (Drosophila) (CNIH4)
22	TK1	thymidine kinase 1, soluble (TK1)
23	MAFB	v-maf musculoaponeurotic fibrosarcoma oncogene homolog B (avian)
24	PPP1CB	protein phosphatase 1, catalytic subunit, beta isoform (PPP1CB), transcript variant 3
25	DNTTIP2	deoxynucleotidyltransferase, terminal, interacting protein 2 (DNTTIP2)
26	ARID4B	AT rich interactive domain 4B (RBP1-like) (ARID4B), transcript variant 1
27	SMARCC2	SWI/SNF related, matrix associated, actin dependent regulator of chromatin, subfamily c,
28	PRO1386	PRO1386 protein
29	TRIOBP	TRIO and F-actin binding protein (TRIOBP), transcript variant 1
30	VARS	valyl-tRNA synthetase
31	ITGA5	integrin, alpha 5 (fibronectin receptor, alpha polypeptide)
32	TERF1	telomeric repeat binding factor (NIMA-interacting) 1 (TERF1), transcript variant 2
33	PURA	purine-rich element binding protein A (PURA)
34	TUBA1B	tubulin, alpha 1b
35	SNRPE	small nuclear ribonucleoprotein polypeptide E
36	RRAGD	Ras-related GTP binding D
37	VWF	von Willebrand factor
39	GLRX3	glutaredoxin 3 (GLRX3)
40	ILF2	interleukin enhancer binding factor 2, 45 kDa

The analysis comparing gene sets in liver tissues from HCV-related non-HCC tissue and controls identified 151 genes differentially expressed. Among them, 127 were shown to be up-regulated and 24 down-regulated in HCV-related non-HCC liver tissues (Figure 3B). The first 40 genes showing the highest fold of up-regulation are listed in Table 2.

**Table 2:** The first 40 up-regulated genes in HCV-related non-HCC counterpart

**N°**	**Gene Name**	**Description**
1	NMNAT3	nicotinamide nucleotide adenylyltransferase 3 (NMNAT3).
2	OASL	2'-5'-oligoadenylate synthetase-like (OASL), transcript variant 2
3	TMPRSS3	transmembrane protease, serine 3 (TMPRSS3), transcript variant C
4	MFSD7	major facilitator superfamily domain containing 7 (MFSD7)
5	AEBP1	AE binding protein 1 (AEBP1), mRNA.
6	UBD	ubiquitin D (UBD)
7	S100A4	S100 calcium binding protein A4 (S100A4), transcript variant 1
8	C1orf151	chromosome 1 open reading frame 151 (C1orf151)
9	CRIP1	Cysteine-rich protein 1 (intestinal)
10	ASCC3	activating signal cointegrator 1 complex subunit 3
11	ZNF271	zinc finger protein 271 (ZNF271), transcript variant 2
12	ANXA4	annexin A4 (ANXA4)
13	NMI	N-myc (and STAT) interactor (NMI)
14	UBE2L6	ubiquitin-conjugating enzyme E2L 6 (UBE2L6), transcript variant 1
15	B2 M	beta-2-microglobulin (B2 M)
16	HLA-F	Major histocompatibility complex, class I, F
17	PSMB9	Proteasome (prosome, macropain) subunit, beta type, 9
18	TAP1	transporter 1, ATP-binding cassette, sub-family B (MDR/TAP)
19	PSME2	proteasome (prosome, macropain) activator subunit 2 (PA28 beta)
20	IFI16	interferon, gamma-inducible protein 16
21	IFI27	interferon, alpha-inducible protein 27
22	ARHGAP9	Rho GTPase activating protein 9
23	RABGAP1L	RAB GTPase activating protein 1-like
24	TNK1	tyrosine kinase, non-receptor
25	DEF6	differentially expressed in FDCP 6 homolog (mouse)
26	BTN3A3	butyrophilin, subfamily 3, member A3
27	RPS6KA1	ribosomal protein S6 kinase, 90 kDa, polypeptide 1
28	CD24	CD24 molecule
29	PARP10	poly (ADP-ribose) polymerase family, member 10
30	APOL3	apolipoprotein L, 3 (APOL3), transcript variant alpha/d
31	STAT	signal transducer and activator of transcription 1, 91 kDa
32	ANKRD10	Ankyrin repeat domain 10
33	CKB	creatine kinase, brain (CKB)
34	H2AFZ	H2A histone family, member Z
35	PSMB9	proteasome (prosome, macropain) subunit, beta type, 9
36	RARRES3	retinoic acid receptor responder (tazarotene induced) 3
37	RGS10	regulator of G-protein signaling 10 (RGS10), transcript variant 2
38	TUBB	tubulin, beta
39	NOL3	nucleolar protein 3 (apoptosis repressor with CARD domain)
40	CD7	CD74 molecule, major histocompatibility complex, class II invariant chain

The analysis comparing gene sets in liver tissues from HCV-related HCC and HCV-related non-HCC counterpartidentified 383 genes differentially expressed. Among them, 83 were shown to be up-regulated and 300 down-regulated in HCV-related HCC liver tissues (Figure 3C). The first 40 genes showing the highest fold of up-regulation are listed in Table 3.

**Table 3:** The first 40 up-regulated genes in HCV-related HCC

**N°**	**Gene Name**	**Description**
1	CAPG	capping protein (actin filament), gelsolin-like
2	OCC-1	PREDICTED: misc_RNA (OCC-1)
3	EED	embryonic ectoderm development (EED), transcript variant 1
4	RPLP0	ribosomal protein, large, P0 (RPLP0), transcript variant 1
5	RPLP0P2	ribosomal protein, large, P0 pseudogene 2
6	AP1S2	adaptor-related protein complex 1, sigma 2 subunit
7	RRAGD	Ras-related GTP binding D (RRAGD)
8	PFDN4	prefoldin subunit 4 (PFDN4)
9	CCDC104	coiled-coil domain containing 104 (CCDC104)
10	C7orf28B	chromosome 7 open reading frame 28B
11	PSIP1	PC4 and SFRS1 interacting protein 1 (PSIP1), transcript variant 2.
12	LPCAT1	lysophosphatidylcholine acyltransferase 1
13	FSCN3	fascin homolog 3, actin-bundling protein, testicular
14	RAB24	RAB24, member RAS oncogene family
15	ZNF446	zinc finger protein 446 (ZNF446)
16	SEC11B	PREDICTED: SEC11 homolog B (S. cerevisiae)
17	ZNF586	zinc finger protein 586 (ZNF586)
18	SCNM1	sodium channel modifier 1
19	SF3A1	splicing factor 3a, subunit 1, 120 kDa
20	RUFY1	RUN and FYVE domain containing 1
21	TRIM55	tripartite motif-containing 55
22	GOLGA4	golgi autoantigen, golgin subfamily a
23	GPATCH4	G patch domain containing 4 (GPATCH4), transcript variant 1
24	THOP1	thimet oligopeptidase 1
25	TUBB2C	tubulin, beta 2C (TUBB2C)
26	PHLDB3	Pleckstrin homology-like domain, family B
27	FAM104A	family with sequence similarity 104, member A
28	FASTK	Fas-activated serine/threonine kinase
29	EIF2AK4	eukaryotic translation initiation factor 2 alpha kinase 4
30	ZFP41	ZFP41--zinc finger protein 41 homolog (mouse)
31	PRKRIP1	PRKR interacting protein 1 (IL11 inducible)
32	DSTN	destrin (actin depolymerizing factor)
33	PHIP	pleckstrin homology domain interacting protein (PHIP)
34	NUCKS1	nuclear casein kinase and cyclin-dependent kinase substrate 1
35	TNRC8	Trinucleotide repeat containing 8
36	CCDC132	coiled-coil domain containing 132
37	EPRS	glutamyl-prolyl-tRNA synthetase
39	HIST1H4C	histone cluster 1, H4c
40	CDCA8	cell division cycle associated 8

### Ingenuity pathway analysis

**Figure 4:**
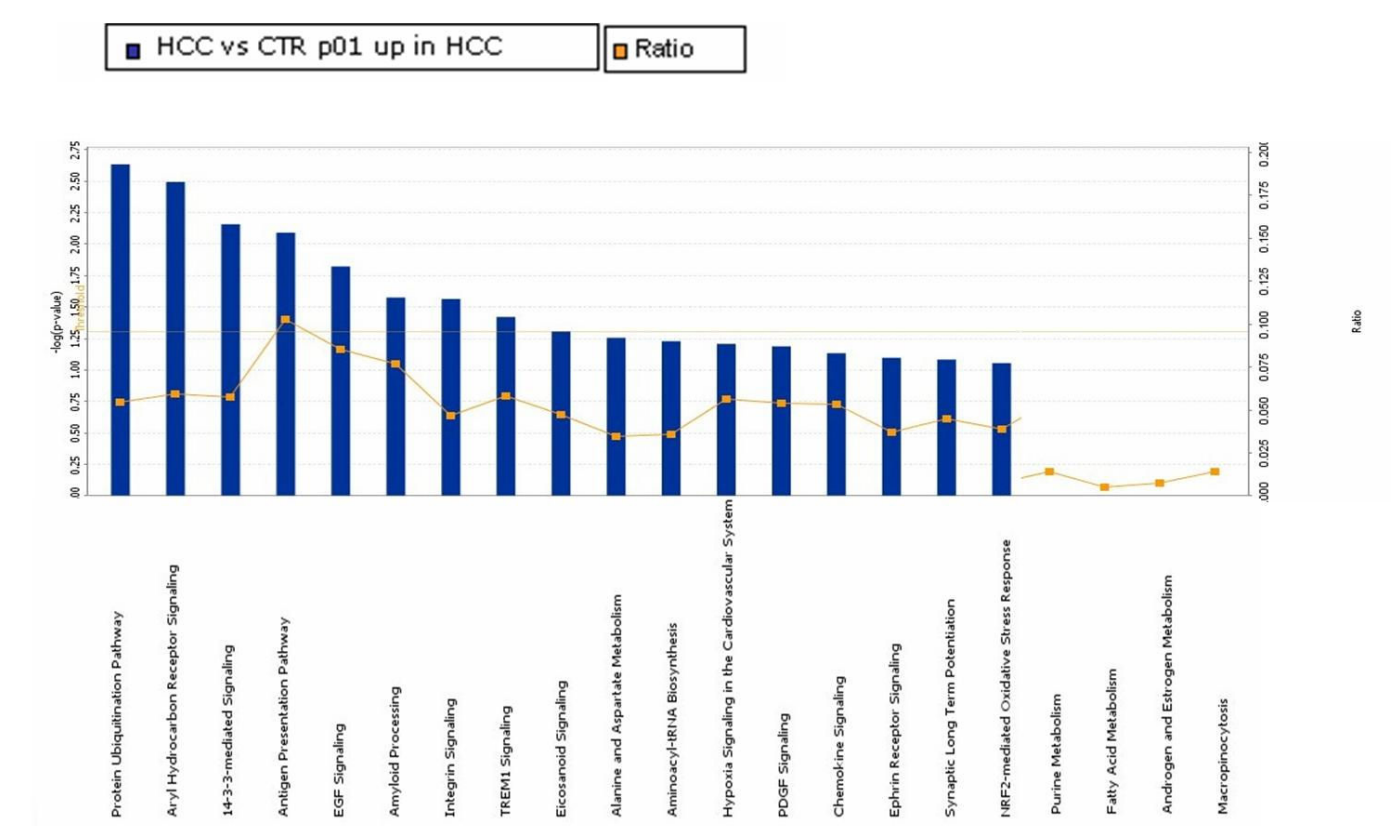
**Significant pathways at the nominal 0.01 level of the unpaired Student's *t*-test**. The human pathway lists determined by "Ingenuity System Database" in HCV-related HCC samples.

**Figure 5:**
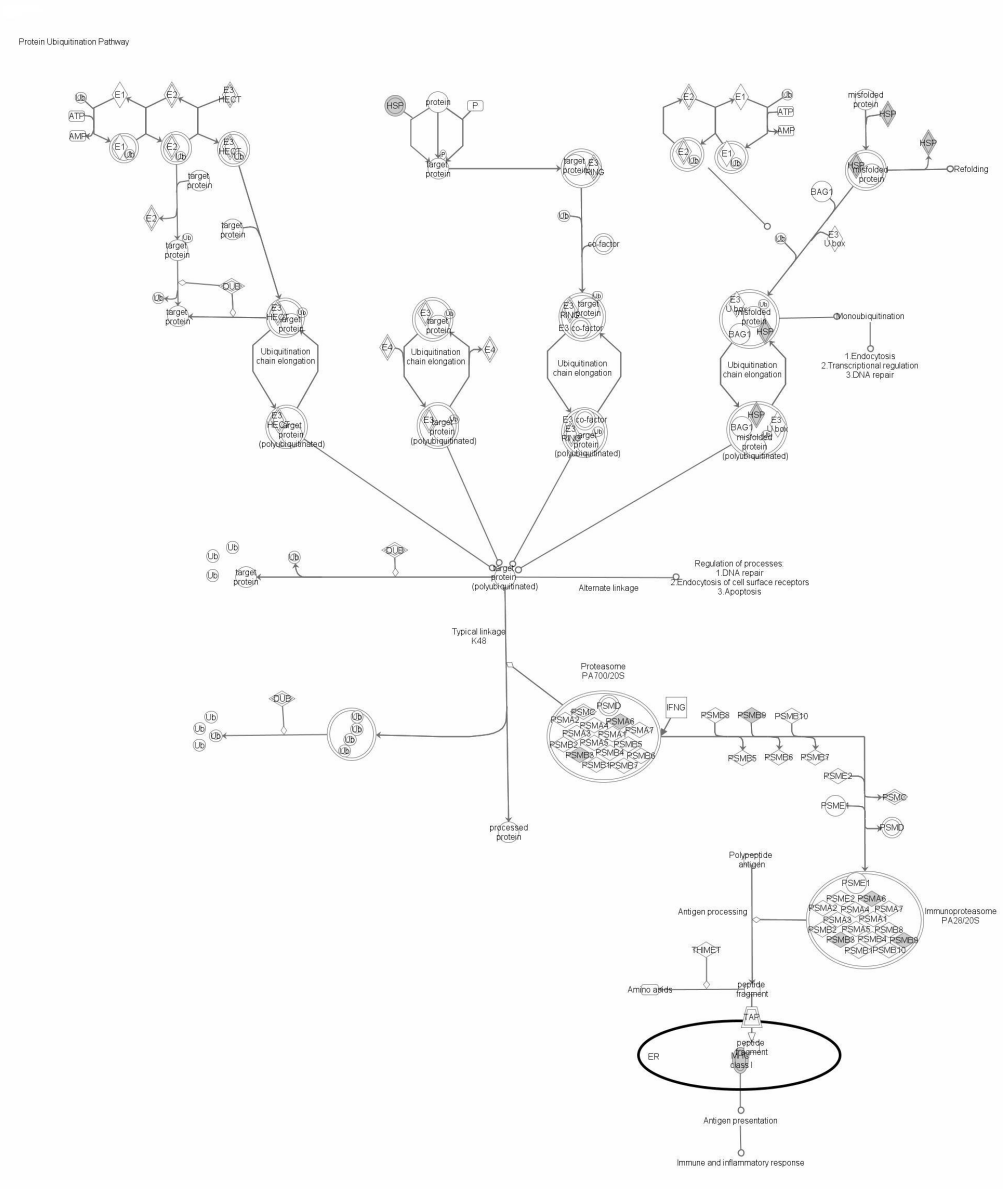
**Significant pathways at the nominal 0.01 level of the unpaired Student's *t*-test**. The 1 top-scoring pathway of genes upregulated IPA image.

**Figure 6:**
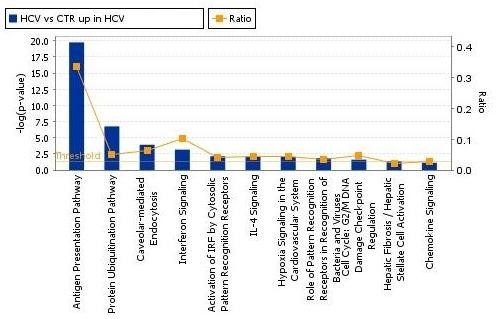
**Significant pathways at the nominal 0.001 level of the unpaired Student's *t*-test**. The human pathway lists determined by "Ingenuity System Database" in HCV-related non-HCC samples.

**Figure 7:**
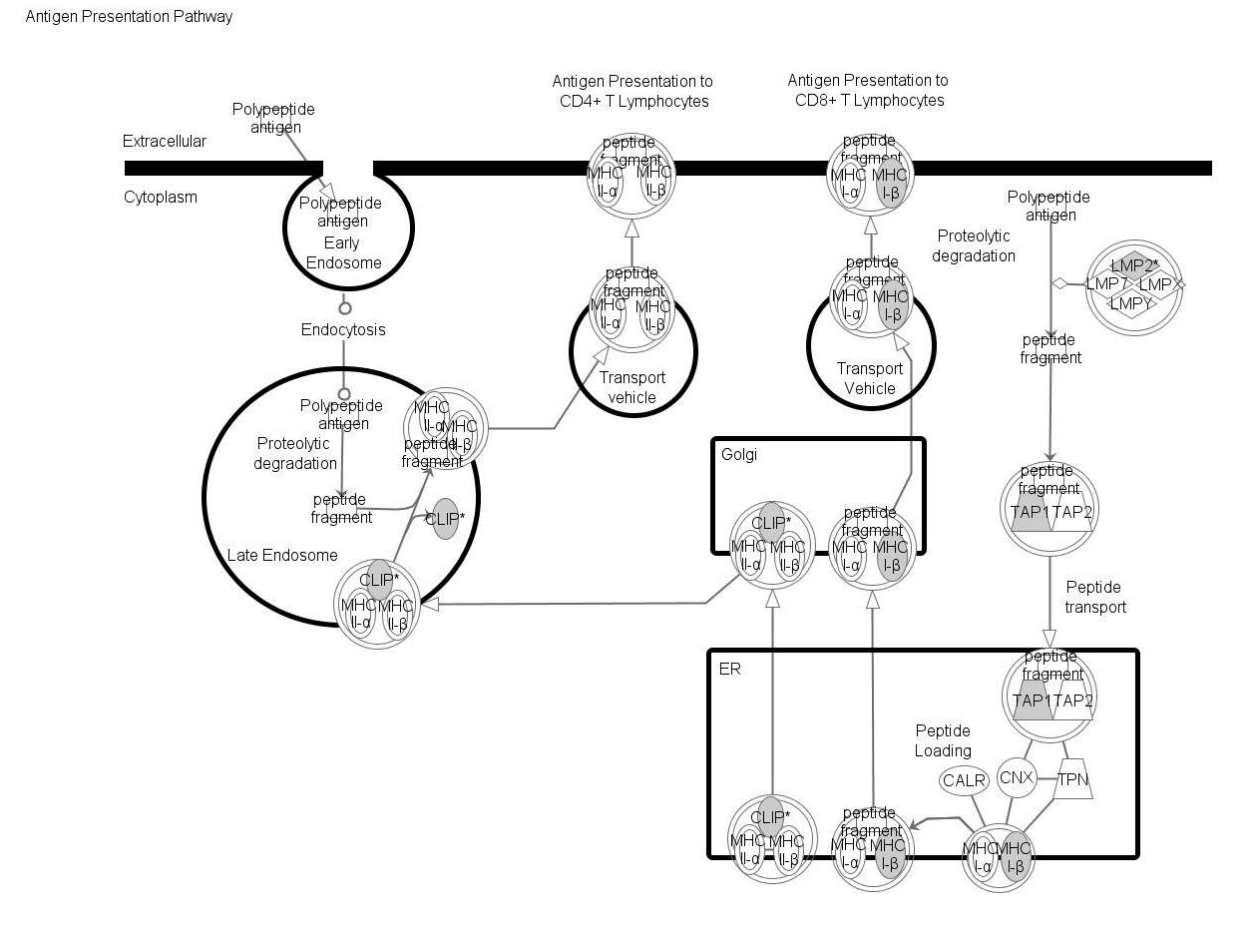
**Significant pathways at the nominal 0.001 level of the unpaired Student's *t*-test**. The 1 top-scoring pathway of genes upregulated IPA image.

The pathway analysis was performed including the genes found up-regulated in the supervised comparisons, using the gene set expression comparison kit implemented in BRB-Array- Tools. The human pathway lists determined by "Ingenuity System Database" was selected. Significance threshold of *t*-test was set at 0.001. Samples from HCV-related non-HCC liver tissue showed strong up-regulation of genes involved in Antigen Presentation, Protein Ubiquitination, Interferon signaling, IL-4 signaling, Bacteria and Viruses cell cycle and chemokine signaling pathways. Samples from HCV-related HCC showed strong up-regulation of genes involved in Metabolism, Aryl Hydrocarbon receptor signaling, 14-3-3 mediated signaling and protein Ubiquitination pathways. Significant pathways were listed respectively in Figures 4, 5, 6 and 7.

## Discussion

The pathogenetic mechanisms leading to HCC development in HCV chronic infection are not yet fully elucidated. In particular, besides inducing liver tissue inflammation and regeneration, which ultimately may result in cellular transformation and HCC development, HCV may play a more direct oncogenic activity inducing an altered expression of cellular genes. To this aim, global gene expression profile can identify specific genes differentially expressed and provide powerful insights into mechanisms regulating the transition from pre-neoplastic to fully blown neoplastic proliferation [23,24].

In the present study, the differential gene expression was evaluated by microarray analysis on liver tissues obtained from fourteen HCV-positive HCC patients and seven HCV-negative control patients. In particular, from each of the HCV-positive HCC patients, a pair of liver biopsies from HCC nodule and non-HCC non adjacent counterpart were surgically excised.

The unsupervised analysis didn't show a clear separation of samples from the 3 different groups (HCV-related HCC, their non-HCC counterpart, as well as control patients), suggesting the lack of a clear-cut distinct gene signature pattern. Nevertheless, normal control samples, with the exception of CTR#76 sample, grouped in a single cluster close to samples from HCV-related paired non-HCC samples. The latter, in fact, comprise several non-HCC pathological stages including dysplastic, not fully transformed lesions, representing pre-neoplastic step in the progression to HCC and should still retain a gene signature pattern closer to normal than to transformed cell physiology. On the contrary, the unsupervised analysis including only one of the HCV-related liver tissues (HCC or non-HCC counterpart) and normal controls showed a clear-cut segregation of the pathological from the control cluster, indicating the identification of specific gene signature patterns peculiar to the HCV-related pre-neoplastic (non-HCC) and neoplastic (HCC) tissues compared to normal controls.

A supervised analysis was performed by pairwise comparison between samples of the three groups analyzed in the present study. The results indicated that the HCV-related HCC liver tissues showed 825 genes differentially expressed compared to controls, of which 465 were up-regulated and 360 down-regulated. The HCV-related non-HCC liver tissues showed 151 genes differentially expressed compared to controls, of which 127 were up-regulated and 24 down-regulated. The HCV-related HCC liver tissues showed 383 genes differentially expressed compared to HCV-related non-HCC counterpart, of which 83 were up-regulated and 300 down-regulated. In each of these independent class comparison analysis, the differentially expressed genes were selected based on a 3-fold difference at a significance *p*-value < 0.01.

The up-regulated genes identified within the individual class comparison analysis were further evaluated and classified by a pathway analysis, according to the "Ingenuity System Database".

The genes up-regulated in samples from HCV-related HCC are classified in metabolic pathways, and the most represented are the Aryl Hydrocarbon receptor signaling (AHR) and, protein Ubiquitination pathways, which have been previously reported to be involved in cancer, and in particular in HCC, progression.

The Aryl Hydrocarbon receptor signal transduction Pathway (AHR) is involved in the activation of the cytosolic aryl hydrocarbon receptor by structurally diverse xenobiotic ligands (including dioxin, and polycyclic or halogenated aromatic hydrocarbons) and mediating their toxic and carcinogenic effects [25,26]. More recently AHR pathway has been shown to be involved in apoptosis, cell cycle regulation, mitogen-activated protein kinase cascades [27]. In particular, studies on liver tumor promotion have shown that dioxin-induced AHR activation mediates clonal expansion of initiated cells by inhibiting apoptosis and bypassing AHR-dependent cell cycle arrest [28]. Furthermore, it has been shown that changes in mRNA expression of specific genes in the AHR pathway are linked to progression of HCV-associated hepatocellular carcinoma [29]. Moreover, the HCV-induced AHR signal transduction pathway, could be directly involved in the increased severity of hepatic lesions in patients with chronic hepatitis C induced by smoking [30,31].

The ubiquitin and ubiquitin-related proteins of the ubiquitination pathway play instrumental roles in cell-cycle regulation [32] as well as cell death/apoptosis [33] through modification of target proteins. In particular, ubiquitin-like proteins, i.e. FAT10, has been reported to bind non-covalently to the human spindle assembly checkpoint protein, MAD2 [34], which is responsible for maintaining spindle integrity during mitosis [35] and whose inhibited function has been associated with chromosomal instability [36,37]. Moreover, FAT10 overexpression has been previously shown in hepatocellular carcinoma [38].

The genes up-regulated in samples from HCV-related non-HCC tissue are classified in several pathways prevalently associated to inflammation and native/adaptive immunity and most of the overexpressed genes belong to the Antigen Presentation pathway. Considering the chronic HCV infection, these result could be unexpected and contradictory, since a reduced native and/or adaptive specific immune response would represent a very much favorable environment for the virus. Nevertheless, these findings, which confirm also a recent report by others [39], could explain the generic massive inflammation and immunopathological tissue damage characteristic of HCV-related cirrhosis [40].

In this study, informative data on the global gene expression pattern in HCV-related HCC as well as HCV-related non-HCC counterpart liver tissues have been obtained compared to normal controls. These data, which need further confirmation studies on a larger set of samples and also at protein level, may be extremely helpful for the identification of exclusive activation markers to characterize gene expression programs associated with progression of HCV-related lesions to HCC.

## Competing interests

The authors declare that they have no competing interests.

## Authors' contributions

FMB, FI, MLT and FMM were responsible for the overall planning and coordination of the study. AW and LB were involved in the data analysis; VDG and EW were involved in genetic analyses. FI was involved in the patients enrollment and liver sample collection. VDG and AM were responsible for specimen processing and RNA analysis. VDG and FMB compiled and finalized the manuscript. All authors read and approved the final manuscript.

## References

[B1] El-Serag HB, Mason AC (1999). Rising incidence of hepatocellular carcinoma in the United States. N Engl J Med.

[B2] Davila JA, Petersena NJ, Nelson HA, El-Serag HB (2003). Geographic variation within the United States in the incidence of hepatocellular carcinoma. J Clin Epidemiol.

[B3] El-Serag HB (2002). Hepatocellular carcinoma and hepatitis C in the United States. Hepatology.

[B4] Romeo R, Colombo M (2002). The natural history of hepatocellular carcinoma. Toxicology.

[B5] Block TM, Mehta AS, Fimmel CJ, Jordan R (2003). Molecular viral oncology of hepatocellular carcinoma. Oncogene.

[B6] Buendia MA (1998). Hepatitis B viruses and cancerogenesis. Biomed Pharmacother.

[B7] Davis GL, Albright JE, Cook SF, Rosenberg DM (2003). Projecting future complications of chronic hepatitis C in the United States. Liver Transpl.

[B8] Colombo M (1998). The role of hepatitis C virus in hepatocellular carcinoma. Recent Results Cancer Res.

[B9] Ohata K, Hamasaki K, Toriyama K, Matsumoto K, Saeki A, Yanagi K (2003). Hepatic steatosis is a risk factor for hepatocellular carcinoma in patients with chronic hepatitis C virus infection. Cancer.

[B10] Brunt EM (2004). Nonalcoholic steatohepatitis. Semin Liver Dis.

[B11] Davila JA, Morgan RO, Shaib Y, McGlynn KA, El-Serag HB (2005). Diabetes increases the risk of hepatocellular carcinoma in the United States: a population based case control study. Gut.

[B12] Fusco M, Girardi E, Piselli P, Palombino R, Polesel J, Maione C (2008). Epidemiology of viral hepatitis infections in an area of southern Italy with high incidence rates of liver cancer. Eur J Cancer.

[B13] Schafer DF, Sorrell MF (1999). Hepatocellular carcinoma. Lancet.

[B14] Levrero M (2006). Viral hepatitis and liver cancer: the case of hepatitis C. Oncogene.

[B15] Macdonald GA, Greenson JK, Saito K, Cherian SP, Appelman HD, Boland CR (1998). Microsatellite instability and loss of heterozygosity at DNA mismatch repair gene loci occurs during hepatic carcinogenesis. Hepatology.

[B16] Blum HE, Moradpour D (2002). Viral pathogenesis of hepatocellular carcinoma. J Gastroenterol Hepatol.

[B17] Iizuka N, Oka M, Yamada-Okabe H, Mori N, Tamesa T, Okada T (2002). Comparison of gene expression profiles between hepatitis B virus- and hepatitis C virus-infected hepatocellular carcinoma by oligonucleotide microarray data on the basis of a supervised learning method. Cancer Res.

[B18] Okabe H, Satoh S, Kato T, Kitahara O, Yanagawa R, Yamaoka Y (2001). Genome-wide analysis of gene expression in human hepatocellular carcinomas using cDNA microarray: identification of genes involved in viral carcinogenesis and tumor progression. Cancer Res.

[B19] Shirota Y, Kaneko S, Honda M, Kawai HF, Kobayashi K (2001). Identification of differentially expressed genes in hepatocellular carcinoma with cDNA microarrays. Hepatology.

[B20] Wang E, Miller LD, Ohnmacht GA, Liu ET, Marincola FM (2000). High-fidelity mRNA amplification for gene profiling. Nat Biotechnol.

[B21] Eisen MB, Spellman PT, Brown PO, Botstein D (1998). Cluster analysis and display of genome-wide expression patterns. Proc Natl Acad Sci USA.

[B22] Ross DT, Scherf U, Eisen MB, Perou CM, Rees C, Spellman P (2000). Systematic variation in gene expression patterns in human cancer cell lines. Nat Genet.

[B23] Nacht M, Ferguson AT, Zhang W, Petroziello JM, Cook BP, Gao YH (1999). Combining serial analysis of gene expression and array technologies to identify genes differentially expressed in breast cancer. Cancer Res.

[B24] Sanchez-Carbayo M, Socci ND, Lozano JJ, Li W, Charytonowicz E, Belbin TJ (2003). Gene discovery in bladder cancer progression using cDNA microarrays. Am J Pathol.

[B25] Safe S (2001). Molecular biology of the Ah receptor and its role in carcinogenesis. Toxicol Lett.

[B26] Okey AB (2007). An aryl hydrocarbon receptor odyssey to the shores of toxicology: the Deichmann Lecture, International Congress of Toxicology-XI. Toxicol Sci.

[B27] Puga A, Ma C, Marlowe JL (2009). The aryl hydrocarbon receptor cross-talks with multiple signal transduction pathways. Biochem Pharmacol.

[B28] Bock KW, Kohle C (2005). Ah receptor- and TCDD-mediated liver tumor promotion: clonal selection and expansion of cells evading growth arrest and apoptosis. Biochem Pharmacol.

[B29] Tsunedomi R, Iizuka N, Hamamoto Y, Uchimura S, Miyamoto T, Tamesa T (2005). Patterns of expression of cytochrome P450 genes in progression of hepatitis C virus-associated hepatocellular carcinoma. Int J Oncol.

[B30] Pessione F, Ramond MJ, Njapoum C, Duchatelle V, Degott C, Erlinger S (2001). Cigarette smoking and hepatic lesions in patients with chronic hepatitis C. Hepatology.

[B31] Hezode C, Lonjon I, Roudot-Thoraval F, Mavier JP, Pawlotsky JM, Zafrani ES (2003). Impact of smoking on histological liver lesions in chronic hepatitis C. Gut.

[B32] Jentsch S, Pyrowolakis G (2000). Ubiquitin and its kin: how close are the family ties?. Trends Cell Biol.

[B33] Jesenberger V, Jentsch S (2002). Deadly encounter: ubiquitin meets apoptosis. Nat Rev Mol Cell Biol.

[B34] Liu YC, Pan J, Zhang C, Fan W, Collinge M, Bender JR (1999). A MHC-encoded ubiquitin-like protein (FAT10) binds noncovalently to the spindle assembly checkpoint protein MAD2. Proc Natl Acad Sci USA.

[B35] Shah JV, Cleveland DW (2000). Waiting for anaphase: Mad2 and the spindle assembly checkpoint. Cell.

[B36] Wang X, Jin DY, Wong YC, Cheung AL, Chun AC, Lo AK (2000). Correlation of defective mitotic checkpoint with aberrantly reduced expression of MAD2 protein in nasopharyngeal carcinoma cells. Carcinogenesis.

[B37] Gemma A, Hosoya Y, Seike M, Uematsu K, Kurimoto F, Hibino S (2001). Genomic structure of the human MAD2 gene and mutation analysis in human lung and breast cancers. Lung Cancer.

[B38] Lee CG, Ren J, Cheong IS, Ban KH, Ooi LL, Yong TS (2003). Expression of the FAT10 gene is highly upregulated in hepatocellular carcinoma and other gastrointestinal and gynecological cancers. Oncogene.

[B39] Mas VR, Maluf DG, Archer KJ, Yanek K, Kong X, Kulik L (2009). Genes involved in viral carcinogenesis and tumor initiation in hepatitis C virus-induced hepatocellular carcinoma. Mol Med.

[B40] Schuppan D, Krebs A, Bauer M, Hahn EG (2003). Hepatitis C and liver fibrosis. Cell Death Differ.

